# Fluoride release from two types of fluoride-containing orthodontic adhesives: Conventional versus resin-modified glass ionomer cements—An in vitro study

**DOI:** 10.1371/journal.pone.0247716

**Published:** 2021-02-26

**Authors:** Yasemin Dziuk, Sachin Chhatwani, Stephan C. Möhlhenrich, Sabrina Tulka, Ella A. Naumova, Gholamreza Danesh

**Affiliations:** 1 Department of Orthodontics, Faculty of Health, University of Witten/Herdecke, Witten, Germany; 2 Faculty of Health, Institute for Medical Biometry and Epidemiology, University of Witten/Herdecke, Witten, Germany; 3 Faculty of Health, Department of Biological and Material Sciences in Dentistry, University of Witten/Herdecke, Witten, Germany; Universitat Bern, SWITZERLAND

## Abstract

**Introduction:**

Development of white spot lesions (WSLs) during orthodontic treatment is a common risk factor. Fixation of the orthodontic appliances with glass ionomer cements could reduce the prevalence of WSL’s due to their fluoride release capacities. The purpose of this study was to evaluate differences of fluoride release properties from resin-modified and conventional glass ionomer cements (GICs).

**Methods:**

The resin-modified GICs Fuji ORTHO LC (GC Orthodontics), Meron Plus QM (VOCO), as well as the conventional GICs Fuji ORTHO (GC Orthodontics), Meron (VOCO) and Ketac Cem Easymix (3M ESPE) were tested in this study. The different types of GICs were applied to hydroxyapatite discs according to the manufacturer’s instructions and stored in a solution of TISAB III (Total Ionic Strength Adjustment Buffer III) and fluoride-free water at 37°C. Fluoride measurements were made after 5 minutes, 2 hours, 24 hours, 14 days, 28 days, 2 months, 3 months and 6 months. One factor analysis of variance (ANOVA) was used for the overall comparison of the cumulative fluoride release (from measurement times of 5 minutes to 6 months) between the different materials with the overall level of significance set to 0.05. Tukey’s post hoc test was used for post hoc pairwise comparisons in the cumulative fluoride release between the different materials.

**Results:**

The cumulative fluoride release (mean ± sd) in descending order was: Fuji ORTHO LC (221.7 ± 10.29 ppm), Fuji ORTHO (191.5 ± 15.03 ppm), Meron Plus QM (173.0 ± 5.89 ppm), Meron (161.3 ± 7.84 ppm) and Ketac Cem Easymix (154.6 ± 6.09 ppm) within 6 months. Analysis of variance detected a significant difference in the cumulative fluoride release between at least two of the materials (rounded p-value < 0.001). Pairwise analysis with Tukey’s post hoc test showed a significant difference in the cumulative fluoride release for all the comparisons except M and MPQM (p = 0.061) and KCE and M (p = 0.517).

**Conclusion:**

Fluoride ions were released cumulatively over the entire test period for all products. When comparing the two products from the same company (Fuji ORTHO LC vs. Fuji ORTHO from GC Orthodontics Europe GmbH and Meron Plus QM vs. Meron from VOCO GmbH, Mannheim, Germany), it can be said that the resin-modified GICs have a higher release than conventional GICs. The highest individual fluoride release of all GICs was at 24 hours. A general statement, whether resin-modified or conventional GICs have a higher release of fluoride cannot be made.

## Introduction

Multi-bracket (MB) treatment during orthodontic treatment is commonly associated with the development of white spot lesions (WSL) with an occurrence mostly reported between 24% and 72,9% [1], but can even vary between 2% up to even 97% [2]. In comparison, the prevalence of WSL’s in patients without orthodontic treatment is only between 11% and 24% [3]. Most of the time lateral incisors and canines are affected by WSL’s [4] and usually occur around the orthodontic fixed appliances [5].

White spot lesions are demineralizations that occur due to bacterial plaque activity and metabolism of carbohydrates resulting in an acid release [6], thus demineralize the dental hard tissue [7] and appear as a patch on the surface of the tooth [8]. Bands, brackets and arches increase plaque retention [2], block access to those plaque retaining areas and complicate adequate cleaning [9].

Therefore it is very important to implement and improve an effective remineralization mechanism [6], which counteracts this process, inhibits acid production and improves resistance to demineralization [7].

Due to their caries-preventive effect through the release of fluoride ions, glass ionomer cements are already widely used for the fixation of orthodontic bands [10] and could be an aid in prevention of white spot lesions.

In restorative materials, the fluoride released has an effective zone of 1 mm [11] and can inhibit demineralization up to 7 mm from the edge of the glass ionomer cement [12]. GICs are capable of maintaining a fluoride concentration of 0.03 ppm in oral saliva after one year [13]. Fluoride release rates in the range of 200–300 μg/cm^2^ per month are considered sufficient to inhibit enamel demineralization [14].

Many studies only cover a time period of one day to 2 months [6, 15, 16]. However, there is little to no information on how fluoride release changes, especially in the first hours, after one month of the so-called "early burst" phase and the long-term release in the range of up to 6 months.

The purpose of this study was the evaluation of the continuous fluoride release of different GICs over a period of 6 months and to compare the differences between resin-modified GICs and conventional GICs.

## Materials and method

In the present study, five different GICs were investigated. The first two groups were resin-modified GICs, groups 3 to 5 were made up of conventional GICs. Group 6 represents a compomer and like group 7 served as an untreated control group (Table 1).

**Table 1:** Materials examined in this study.

Group	Product	Material compostion	Company	Lot number	Product application
1	Fuji ORTHO LC (FOLC) (resin-modified GIC)	Liquid: 2-hydroxyethyl methacrylate 25–50%, Tartaric acid 5–10%, 2-Hydroxy-1 and 3-dimethacryloxypropane 1–5%, 7, 7, 9 (or 7, 9, 9)–Trimethyl-4, 13 –dioxo-3, 14-dioxa-5,12-diazahexadecane-1, 16-diylbismethacrylate 1–5%	GC Orthodontics Europe GmbH, Breckerfeld, Germany	Liquid: 1804021	etching: optional surface conditioning:-mixing ratio:1 spoon / 2 drops of liquid (3.0g / 1.0g) processing time: 3min setting time: 5 1/2min light curing time: 40s
				Powder: 1804051	
		Powder: Oxides, silanes			
2	Meron Plus QM (MPQM) (resin-modified GIC)	Fluoroaluminosilicate glass 50–100%, hydroxypropyl methacrylate 10–25%, glycerine dimethacrylate 5–10%, Urethane dimethacrylate 2.5–5%	VOCO GmbH, Mannheim, Germany	1904080	etching:—surface conditioning:- mixing ratio:—processing time: 2min setting time: 4 1/2min light curing time: optional 5–10s
3	Fuji ORTHO (FO) (conventional GIC)	Liquid: 2-hydroxyethyl methacrylate (HEMA) 25–50%, polybasic carboxylic acid 5–10%, urethane dimethacrylate (UDMA) 1–5% Powder: confidential business information	GC Orthodontics Europe GmbH, Breckerfeld, Germany	Liquid: 808031	etching: optional surface conditioning: Fuji Conditioner 20s, rinse, blow and dry mixing ratio: 1 spoon / 2 drops of liquid (3.0g / 1.0g) processing time: 3min setting time:5 1/2min light curing time:-
				Powder: 1808101	
				Conditioner: 1807101	
4	Meron (M) (conventional GIC)	Liquid: L-tartaric acid 10–15%	VOCO GmbH, Mannheim, Germany	Liquid: 1848330	etching:—surface conditioning:—mixing ratio: 1 measuring spoon / 1 drop of liquid (3.0g / 1.0g) processing time: 3min setting time: 5 – 7min light curing time:-
				Powder: 1851411	
		Powder: Fluoroaluminosilicate glass 50–100%, Polyacrylic acid 25–50%			
5	Ketac Cem Easymix (KCE) (conventional GIC)	Liquid: Wasser 80–90%, Weinsäure < 20%	3M ESPE GmbH, Germany	Liquid: 4328913	etching: -surface conditioning: -mixing ratio: 1 measuring unit powder / 2 drops of liquid (3.8g / 1.0g) processing time: 3min setting time: 7min light curing time:-
				Powder: 4303869	
		Pulver: Polycarbonsäure 20–25 Gew-%,Glaspulver 70–80 Gew-%			
6	Ultra Band Lok (UBL) (compomer)	BisGMA 10–30%, Barium Monoxide 3,90–6,51%, 2-Hydroxyethyl Methacrylate 1–5%, 3-(Trimethoxysilyl)propyl-2-Methyl-2-Propenoic Acid 0,26–3,90%, SG-355RG4000CMP3 30–50%	Reliance Orthodontic Products Inc., Itasca, USA	-	etching:-surface conditioning: -mixing ratio:-processing time:-setting time:-light curing time:30s
7	hydroxyapatite discs	hydroxyapatite	HiMed Inc., Old Bethpage, USA	-	not applicable

GIC = glass ionomer cement g = gram/grams s = second/seconds min = minute/minutes.

resin-modified glass ionomer cements (group 1 and 2); conventional glass ionomer cements (group 3–5); control groups (group 6 and 7).

Flouride–free hydroxyapatite discs (HiMed Inc., Old Bethpage, USA) were used as carrier discs (5mm x 2mm) for the test materials. To control the exact amount of material applied an analytical balance was used (analytical balance, Secura CPA124S, Sartorius AG, Goettingen, Germany). On average 0,025g of test material was used on each disc. If there was a notable difference in weight of the test specimen the process had to be repeated.

The test material was applied to the carrier discs according to manufacturer instructions (Table 1). Surface conditioning was only necessary for Fuji Ortho (FO) with Fuji Ortho Conditioner (GC Orthodontics Europe GmbH, Breckerfeld, Germany). No material was applied to the carrier discs of the seventh group.

Sample size consisted of ten test specimens per group. The selection of n = 10 per group was based on previously published studies [17–20].

The storage solution in which the carrier discs were placed contained a TISAB III solution (Total Ionic Strength Adjustment Buffer, Thomas Scientific, Swedesboro, USA) with bi-distilled water (Megro GmbH & Co. KG, Wesel, Germany) with a mixing ratio of 5%:95%.

For measurements, TISAB III was used to maintain a low pH value of 5–5.5 with constant ionic strength and to unmask the fluoride ions of the samples.

The fluoride measurement was performed with a fluoride electrode (Orion 9609BNWP, Thermo Fisher Scientific Inc., Chelmsford, USA). The measurement was repeated five times for each test specimen to prevent fluctuation. Calibration of the electrode is essential to obtain reliable measured values. For this reason, four fluoride standard solutions were prepared with sodium fluoride and diluted TISAB III containing fluoride of 0.38, 3.8, 38 and 380ppm.

Thereby a two-point calibration could be carried out with solutions containing an exact ion concentration. If the measured values deviated by more than 5%, the calibration had to be restarted. The required slope of the electrode in mV mode had to be between 56mV and 60mV. A further solution served additionally as a precision control to ensure consistency of the electrode on each measuring day. This precision control solution was obtained in preliminary tests, in which a pool of cryotubes containing a solution with known fluoride ion concentration was created. They were split up into two separate pools P1 and P2, consisting of a group with low and high amounts of fluoride (P1: 0,6566 ppm, P2: 16,7 ppm).

The 70 test specimens for this study were each kept in an individually labeled cryotube with 1.5ml diluted TISAB III and were stored in an incubator (WTB Binder, Tuttlingen, Germany) at 37°C for the entire duration of the experiment (MELAG Medizintechnik oHG, Berlin, Germany) except for measurement procedures. Fluoride ion measurements were carried out at the following times:

After 5 minutes, 2 hours, 24 hours, 14 days, 4 weeks, 2 months, 3 months and 6 months.

At each measurement time, the carrier discs were placed in new cryotubes containing 1.5ml diluted TISAB III and were replaced in the incubator at 37°C.

After separation of the carrier discs, the old cryotubes were placed in a vortex mixer for 10 seconds and subsequently 500μl of the solution was removed with a pipette and inserted in an empty test tube (4ml) for the measurement of the fluoride ion concentration. This procedure was repeated five times per tube to detect any fluctuations.

The fluoride ion electrode was rinsed with bi-distilled water before and after every contact with the tested solution.

Descriptive analysis for the primary endpoint (cumulative fluoride release) was performed with boxplots and nonparametric location parameters (minimum, first quartile, median, third quartile, maximum) supplemented with arithmetic mean and standard deviation of the cumulative fluoride release for every material.

One factor analysis of variance (ANOVA) was used for the overall comparison of the cumulative fluoride release (from measurement times 5 minutes to 6 months) between the different materials (FOLC, MPQM, FO, M, KCE) as primary statistical analysis of this study with the overall level of significance set to 0.05. As there was no fluoride release for UBLOK and the control these two materials were excluded from the ANOVA (see results section). Levene’s test was used to assess the equality of variances (a p-value greater than 0.05 indicates that the equality of variances cannot be rejected). Quantile-quantile plots (QQ-Plots) will be used for comparing the probability distribution of data with the normal distribution. Tukey’s post hoc test was used for post hoc pairwise comparisons in the cumulative fluoride release between the different materials. Results will be presented with difference in means with standard deviation and adjusted p-values. Additional descriptive analysis for every measuring time is presented with means and line plots. All the statistical analysis of data was done with R Version 3.6.0 (Software R Core Team, 2019).

## Results

After 5 minutes a fluoride ion release could already be observed for FOLC with 2.14 ± 0.47 ppm and KCE with 28.97 ± 1.76ppm.

A rise in fluoride at measuring time after 2 hours ion release was observed for all test materials except for KCE and the control groups. After 24 hours all materials with fluoride release (MPQM, FOLC, FO, M, KCE) had the highest individual fluoride ion release. FO achieved the highest value at this time of measurement with a release of 62.06 ± 13.45 ppm whereas the lowest value was seen for KCE with 31.66 ± 2.48 ppm. Between 24 hours and 14 days, a clinically relevant decrease in fluoride ion release was observed for every material with fluoride release. In the following period from 4 weeks to 6 months a steady fluoride release plateau was established (Fig 2). There were only relatively small fluctuations within the intervals in the individual groups. The highest fluctuations were found for MPQM and the lowest fluctuations for M.

**Fig 1:**
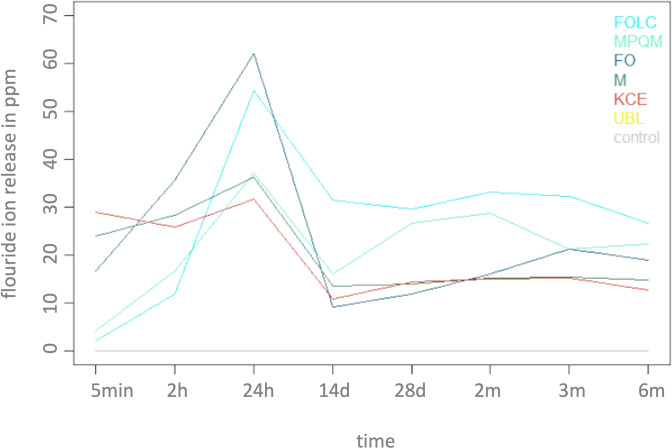
Individual release of fluoride ions (in ppm) at the individual times 5 minutes—6 months of groups 1–7. ppm = parts per million; min = minute/minutes; h = hours; d = days; m = months.

Analysis of the control groups revealed a fluoride ion release of UBL after 5 minutes of 0.046 ± 0.02 ppm while the untreated control group had a delivery of 0.059 ± 0.01 ppm. After 2 hours the untreated control showed a small increase to 0.1 ± 0.25 ppm, with a subsequent decrease to a level of 0.01 ± 0.00 ppm after 24 hours. After 14 days to 6 months changes in fluoride ion release for UBL and the untreated control group remained negligible. Compared to the other materials (MPQM, FOLC, FO, M, the control groups didn’t show any relevant fluoride release (Fig 1).

FOLC and MPQM show a similar pattern of fluoride ion release over time with a peak after 24 hours, declining after 14 days and then building a plateau. This additional analysis was executed by descriptive means. No time dependent statistical tests were performed for the comparison of FOLC and MPQM. But post hoc test in the primary analysis of this study showed a significant cumulative fluoride release over time (p < 0.001) (Fig 1).

Primary analysis was done via one factor (material) analysis of variance for the cumulative fluoride release. The following materials were included in this analysis: FOLC, MPQM, FO, M, KCE. The control material as well as UBLOK were excluded from the analysis of variance due to the missing fluoride release (see above). The normality-assumption of the data was not refused as no qq-plot showed great or systematic deviations from the baseline. A significant difference in total cumulative fluoride ion release between at least two of the investigated materials FOLC, MPQM, FO, M and KCE could be found (p < 0.001).

Following pairwise comparisons with Tukey’s post hoc test showed no significant difference (p > 0.05) in cumulative fluoride release between Meron and Meron Plus QM (Fig 1). All other materials had statistically different cumulative fluoride release in the pairwise comparison (p < 0.001).

The values of conventional GICs peak after 24 hours and drop to lower values after 14 days than those of resin-modified GICs. The pattern of fluoride ion release was comparable for all conventional GICs materials. As this additional analysis was conducted with descriptive statistics no statistical tests were performed for this comparison with regard to FO, M and KCE over time. But pairwise comparison (post hoc Tukey Test in the primary analysis) of conventional and resin-modified glass ionomer cements of the same manufacturer showed a higher cumulative fluoride release from 5 minutes to 6 months in the resin-modified glass ionomer group. Cumulative release was significantly higher when comparing FOLC and FO (p < 0.001). MPQM showed higher cumulative fluoride release in comparison to M but the difference was not statistically significant (p > 0.05).

**Fig 2:**
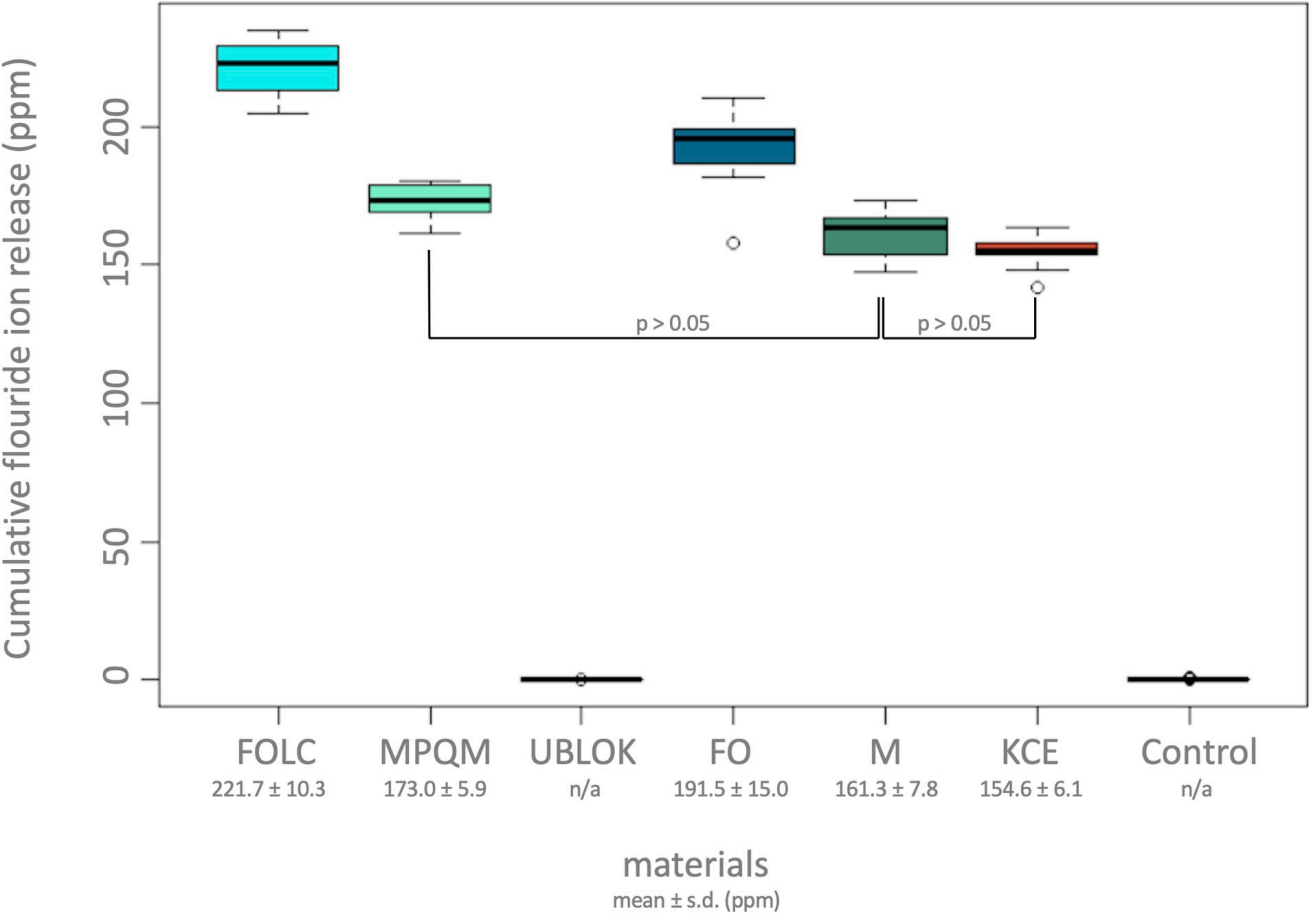
Boxplots of the cumulative fluoride release over all measurement points for all materials (FOLC, MPQM, UBLOK, FO, M, KCE, Control). s.d. = standard deviation; ppm = parts per million; p-value given for Tukey´s post hoc test.

Cumulative fluoride ion release, over all measurement times, of all tested materials can be seen in Fig 2.

## Discussion

Many studies have already investigated the fluoride release of both resin-modified glass ionomer cements and conventional glass ionomer cements but show differing results [6, 21, 22].

This might depend on several different conditions regarding the experimental setup. Factors such as the size of the contact surface to test medium [14], the ratio of powder to liquid during the mixing process and the preparation of the coated surface areas [23] should be taken into account, as well as the decisive influence on fluoride release caused by the length of light-curing time on the used GICs [24].

In artificial saliva, fluoride ion release has shown to be lower than in deionized water, because of its higher saturation caused by calcium and phosphate ions [25]. A varying pH value also influences the speed of fluoride release [26]. In solutions with lower pH values, fluoride is released more frequently [27]. To keep the pH value on a persistent level TISAB III with bi-distilled water was used as storage medium in this study. The usage as storage medium is in concordance with other previously published studies [15, 28, 29].

A lot of studies concerning the fluoride release of fluoride varnishes used hydroxyapatite discs as carrying objects [30–32]. Fluoride release is a three-dimensional process in which every surface area will release ions, as long as there is contact with the surrounding solution. In in-vivo situations GICs have a much smaller contact area to saliva due to the bracket and the adhesive gap. Therefore there is a reduced possibility to release fluoride ions than in comparison to GIC covered hydroxyapatite discs in in-vitro procedures.

The use of fluoride free hydroxyapatite discs as carrier for the GICs in this study minimizes the effective fluoride releasing surface and leads to an overall more realistic fluoride release measurement. Human or bovine teeth as a carrier object would bear the risk of possible higher fluoride release due to additional fluoride ion release from the tooth surfaces itself into the surrounding solution [33] and are therefore not suitable for this process.

Fluoride release is also highly dependent on the temperature at which the test samples were stored during the testing period. At 55°C the fluoride ion release is higher and at 4°C lower as compared to 37°C [34–36]. Due to this effect most studies do not use thermocycling and stored the test specimens at a temperature of 37°C continuously [16, 37, 38]. It has been advocated to use 5–55°C as a thermocycling process for dental materials in the last twenty years [39] and it has been shown that hot food intake can even achieve higher temperatures [40]. For purposes of scientific standardization this study investigates fluoride concentration dynamics in the surrounding solution at 5–55°C.

Fluoride release occurs through three mechanisms: washing off the surface, diffusion through the pores and cracks and mass diffusion from deeper layers [36]. A high release is desirable without changing the physical properties or provoking excessive cement degradation [14]. Apart from the influencing factors of the test setup, the filler composition of GICs, solubility of glass particles and reactivity of powder and liquid determine the amount of released fluoride [41, 42]. Particle size also plays an important role. The smaller the filler particles, the higher is the amount of fluoride released. This is due to an increased surface area created by smaller particles [43].

An increased fluoride release of the resin-modified glass ionomer cements compared to conventional glass ionomer cements might be explained by higher porosity and larger pore size of resin-modified GICs [44–46].

Differences in size of particles or pores of the tested products could explain that a significant difference was found when comparing conventional and resin-modified GIC from the same manufacturer but no statistical significance could be shown in fluoride ion release performance when comparing different manufacturers to each other.

In principle, no general statement can be made by this study as to whether resin-modified or conventional glass ionomer cements release more fluoride as the p-value of Levene’s test was 0.3196 (greater than 0.05) the assumption of equality of variances could not be rejected.

Other studies could also not find a clear difference between these two groups [21, 47].

A comparison in the present study of resin-modified and conventional GICs from the same manufacturer showed a higher cumulative fluoride release of resin-modified GICs. A direct comparison is difficult to determine due to different compositions of the products and is not universally transferable. The potential fluoride release of resin-modified GICs is similar to those of conventional GICs, but are affected by several variables. Such as the formation of complex fluoride compounds, the type and quantity of resin used in the photochemical polymerization reaction and their interaction with polyalkenoic acids [48].

The highest amount of fluoride was released within the first 24 hours [21, 34, 49], which is also determined in this study with the highest individual release being achieved at 24 hours across all groups. A reduction in the amount of fluoride ions released can be observed on the second day. This first phase, which can last up to a month, is called the "early or initial burst" phase [50, 51] and could be confirmed by our findings.

After the initial burst which may occur due to loosely bound fluoride [52] a difference in pattern can be seen in conventional and resin-modified glass ionomer cements (28 days, 2 months). It is stated that a stabilization phase of the released fluoride occurs after 7 to 11 days [53]. In this study, the stabilization phase of the released fluoride was found after 2 weeks. The plateau of fluoride release seems higher for resin modified glass ionomer cements (Fig 1). A possible explanation has been described by Cabral et al. stating that the resin components slow down the acid-based reactions and thereby the ionic matrix is able to release more fluoride over time [54]. Interestingly a minimal amount <0.01ppm of fluoride concentration is sufficient to facilitate remineralization in in vitro studies. The minimum concentration in vivo could not be determined [55]. Therefore the differences in plateaus might have clinical relevance.

As the discs were not coated simultaneously at one single time in this study, it can be assumed that there may be minimal differences in the mixing ratio of powder and liquid for the test materials. The measured values of fluoride ion concentration are in a low range that even with the use of a highly sensitive fluoride, as in this study, accuracy cannot be guaranteed. This could also be one of the reasons why a minimal amount of fluoride release was measured in the control group with hydroxyapatite discs. Contamination of the storage solution as possible error could not be ruled out. Other studies have also shown minimal fluoride release from hydroxyapatite discs serving as a control group [54].

This study has an in-vitro character therefore further research should focus on clinical research in order to validate our findings and their effect on the incidence of WSL under clinical conditions.

## Conclusion

All used products released fluoride ions over the entire test period. The highest release was achieved with Fuji Ortho LC (FOLC), the lowest release was achieved with Ketac Cem Easymix (KCE). Comparing the resin-modified and conventional GICs from the same manufacturer (FOLC vs FO by GC Orthodontics and MPQM vs M by VOCO), it could be noted that resin-modified GICs showed a higher cumulative fluoride release rate than conventional GICs. But comparing the performance of conventional GIC to resin-modified GIC of different manufacturers to one another revealed no statistical significance. Therefore a valid statement for higher fluoride release capacity in general for resin-modified GIC can not be supported.

## Supporting information

S1 File (DOCX)
