# Supplementary material for: Fluoride release from two types of fluoride-containing orthodontic adhesives: Conventional versus resin-modified glass ionomer cements—An in vitro study
Source: PLoS One. 2021 Feb 26;16(2):e0247716. doi: 10.1371/journal.pone.0247716 (PMC7909673; doi:10.1371/journal.pone.0247716)
Supplement: S1 File — (DOCX) [file pone.0247716.s001.docx]

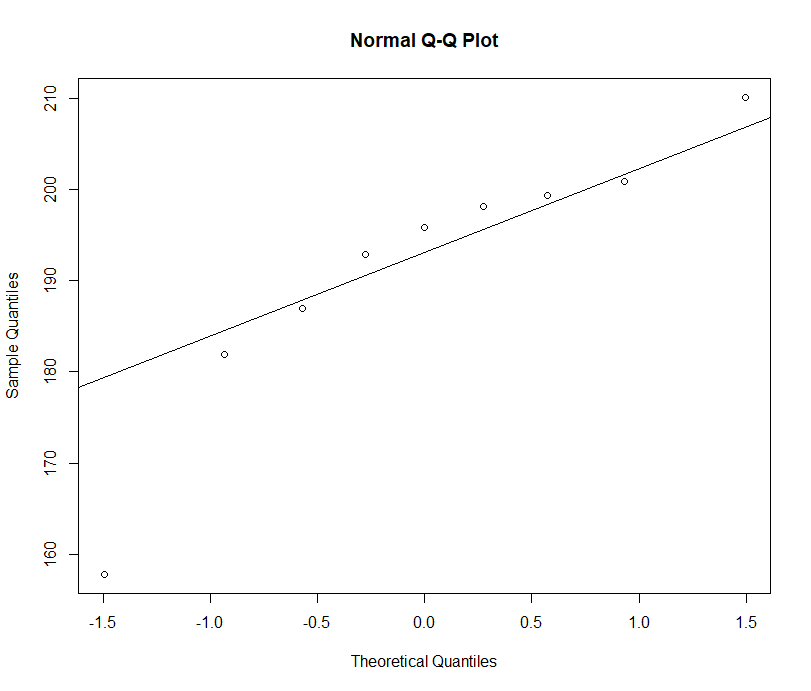


Normal QQ-Plot for Fuji ORTHO


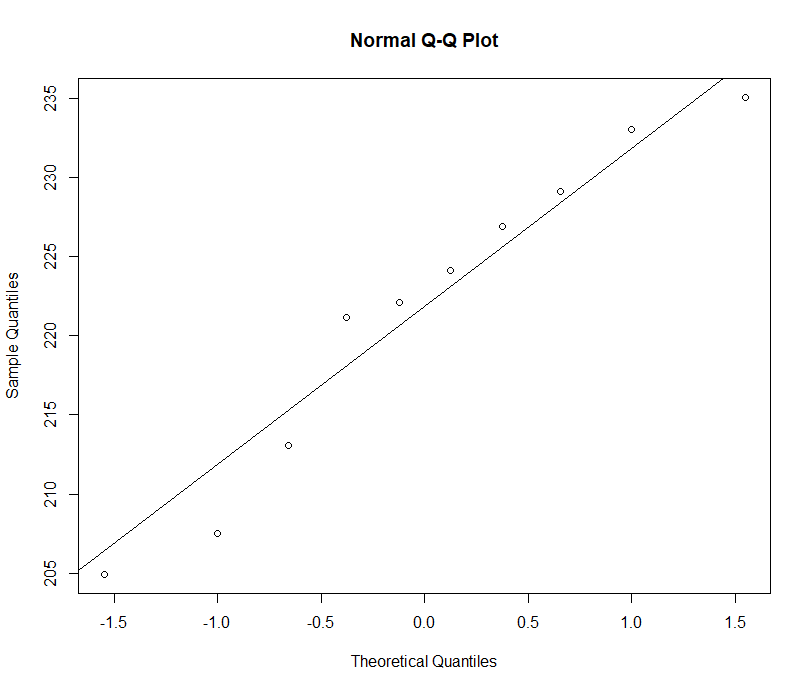


Normal QQ-Plot for Fuji ORTHO LC


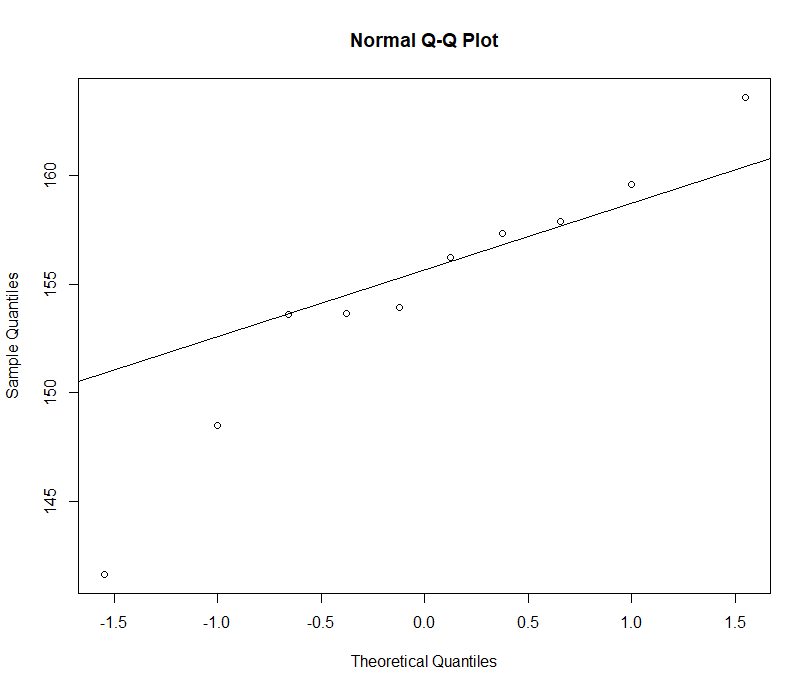


Normal QQ-Plot for Ketac Cem Easymix


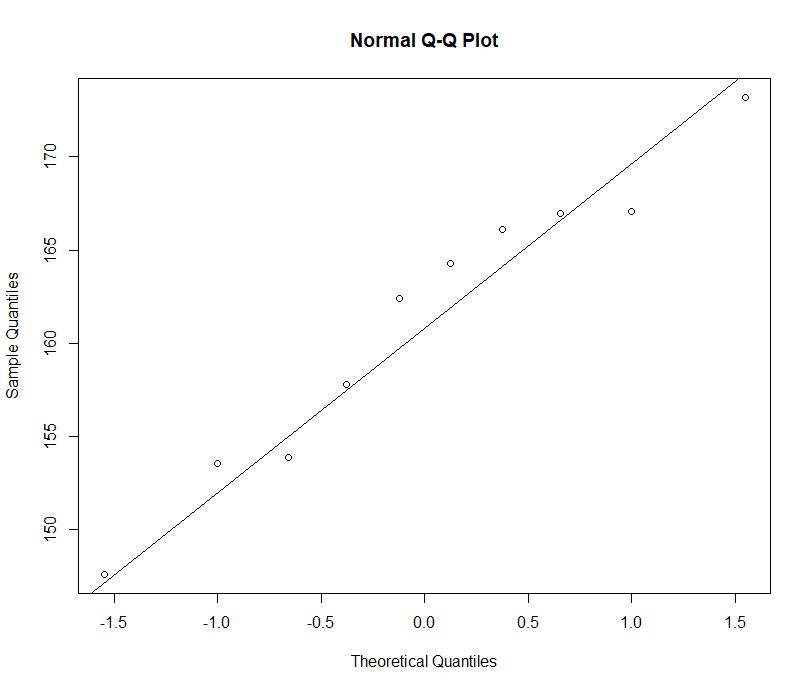


Normal QQ-Plot for Meron


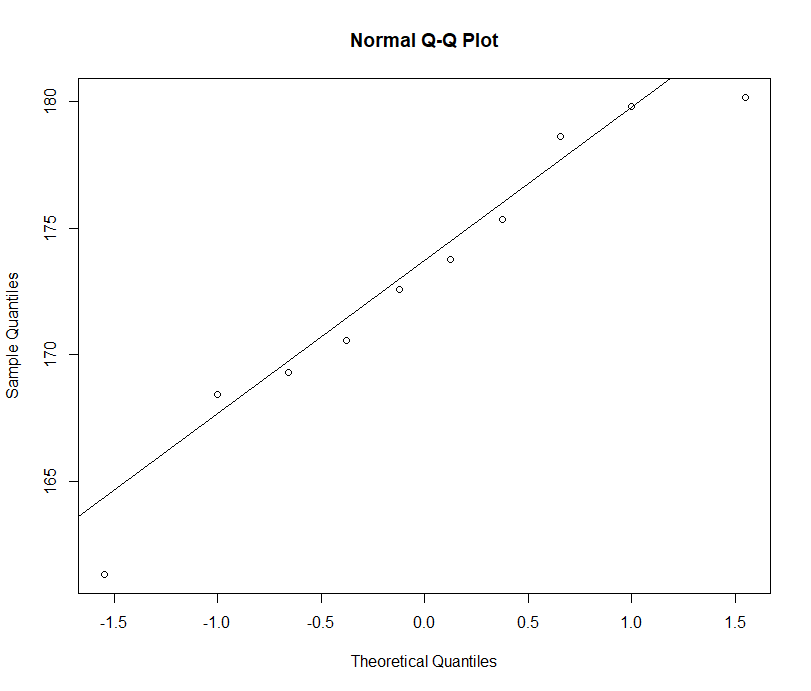


Normal QQ-Plot for Meron Plus QM
